# Primary adult renal pelvic soft tissue giant cell tumor: A rare case report

**DOI:** 10.1097/MD.0000000000050142

**Published:** 2026-08-07

**Authors:** Yong Ou, Ming-xian Chen, Yun Hu, Kai Wang, Zhi-gang Chen, Hua-kang Wang, Mao-lin Ye, Hua Yang, Huasong Hua, Zhong Zheng

**Affiliations:** a Department of Urology, Xichang People’s Hospital, Xichang, Sichuan, China.

**Keywords:** case report, renal pelvis, soft-tissue giant cell tumor

## Abstract

**Rationale:**

Pyelonephric giant cell tumor of soft tissue represents an exceptionally uncommon neoplasm of the urinary tract with limited malignant potential. It typically follows a benign clinical trajectory, with distant metastases occurring infrequently; reports of aggressive disease culminating in lethal multiorgan dissemination are exceedingly rare. The present case seeks to augment the clinical dataset and elucidate the features of its malignant transformation.

**Patient concerns:**

A 72-year-old male presented with a 3-day history of persistent right-sided low back pain, without fever, gross hematuria, or dysuria. Contrast-enhanced abdominal computed tomography imaging revealed wall dilation and hypertrophy of the right renal pelvis-ureteral transitional segment with lipomatous enhancement, measuring approximately 3.3 × 2.7 cm.

**Diagnoses:**

Combined with imaging manifestations and postoperative pathological examination results, the patient was definitively diagnosed with a pyelonephric soft tissue giant cell tumor.

**Interventions:**

The patient underwent radical nephroureterectomy after systematic preoperative evaluation.

**Outcomes:**

Regular postoperative follow-up was performed. Adrenal gland and pulmonary metastatic lesions were detected at 6 months postoperatively. The patient died of continuous tumor progression at 15 months after surgery.

**Lessons:**

This case illustrates that soft tissue giant cell tumor is a rare neoplasm, with renal pelvis soft tissue giant cell tumor being exceedingly uncommon. Diagnosis primarily relies on histopathological examination. Clinicians should include renal pelvis soft tissue giant cell tumor in differential diagnoses. When feasible, en bloc surgical resection is recommended. Postoperative surveillance should be intensified to facilitate early detection of recurrence, as some patients harbor potential relapse, and prognosis remains cautiously guarded.

## 1. Introduction

Giant cell tumor of soft tissue is a rare tumor with low malignant potential. Most lesions are benign and metastasis is rare. Soft tissue giant cell tumors present as multilobulated masses in the skin or subcutaneous tissues, most commonly in the trunk, upper limbs, and proximal lower limbs.^[1]^ Although soft tissue giant cell tumor (GCT-ST) has very similar clinical features and pathological findings to giant cell tumor of bone (GCTB), its exact pathogenesis has not been fully elucidated. Most cases of GCT-ST have a benign course, but rare cases of local recurrence or distant metastasis have been reported.^[2]^ Therefore, complete resection is necessary whenever possible. The presence of giant cell tumors in the kidney needs to be distinguished from giant cell tumors and giant cell urothelial endothelial carcinoma. In this report, we describe a case of primary GCT-ST of the renal pelvis in a patient who presented with pulmonary and adrenal metastases 6 months after surgery, which were considered to be malignant metastases.

## 2. Case report

**Figure 1.**
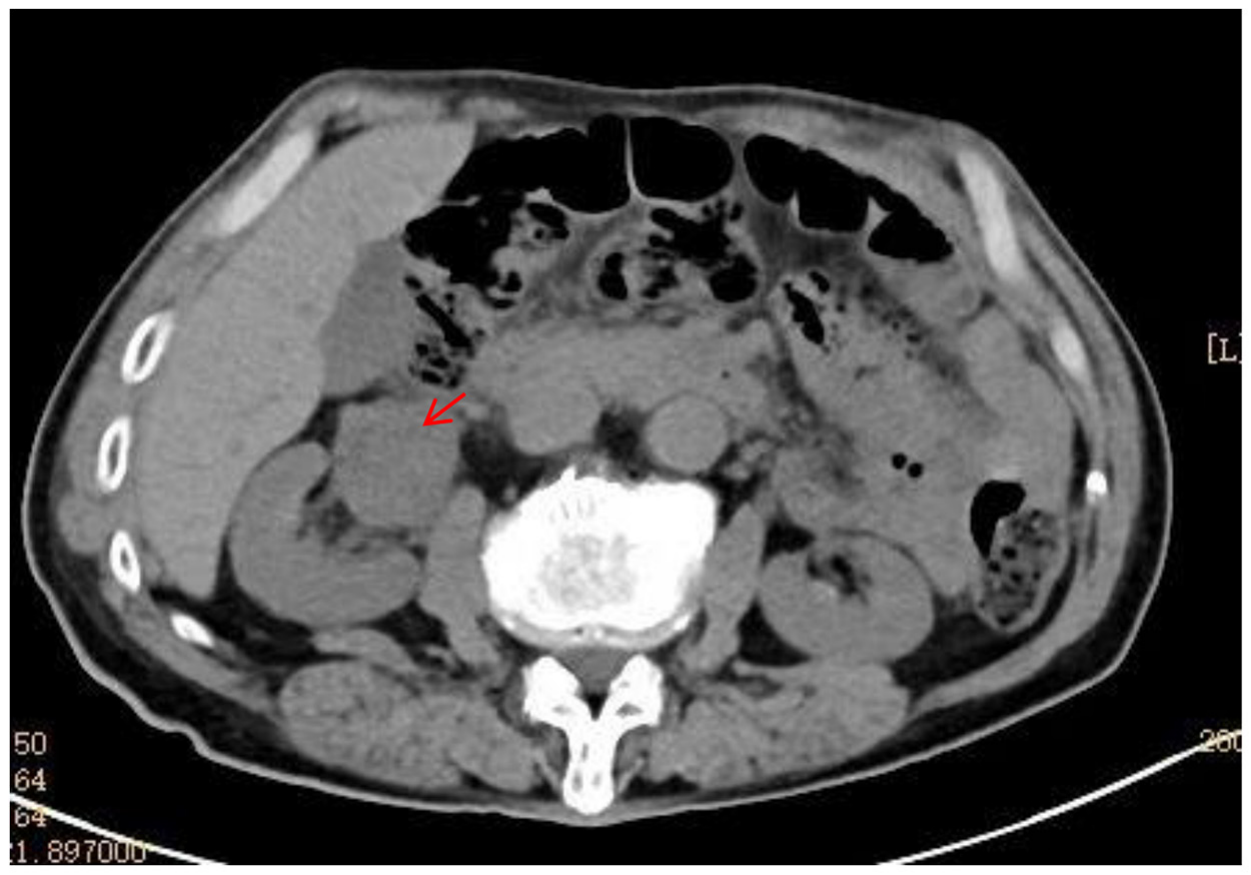
CT scan of the abdomen showing a tumor in the right renal pelvis. CT = computed tomography.

**Figure 2.**
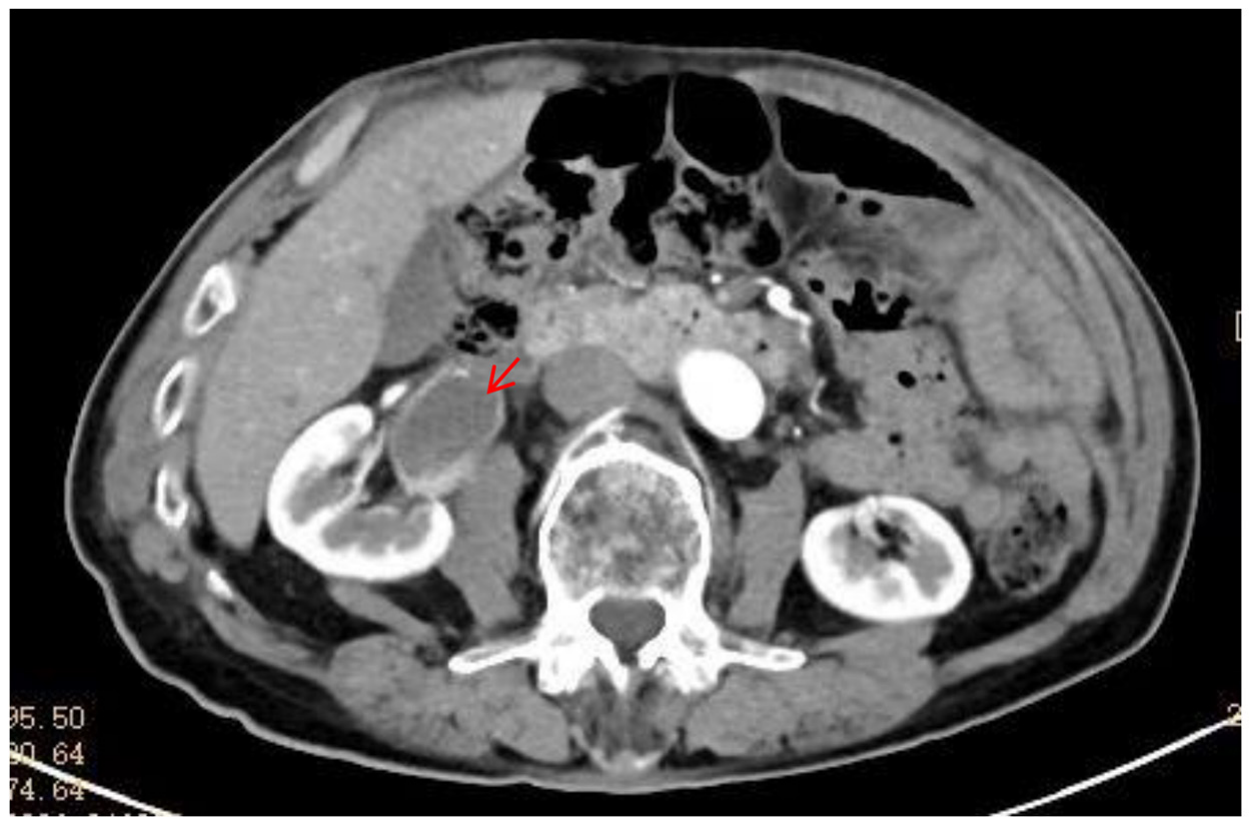
CT scan of the abdomen showing a tumor in the right renal pelvis. CT = computed tomography.

**Figure 3.**
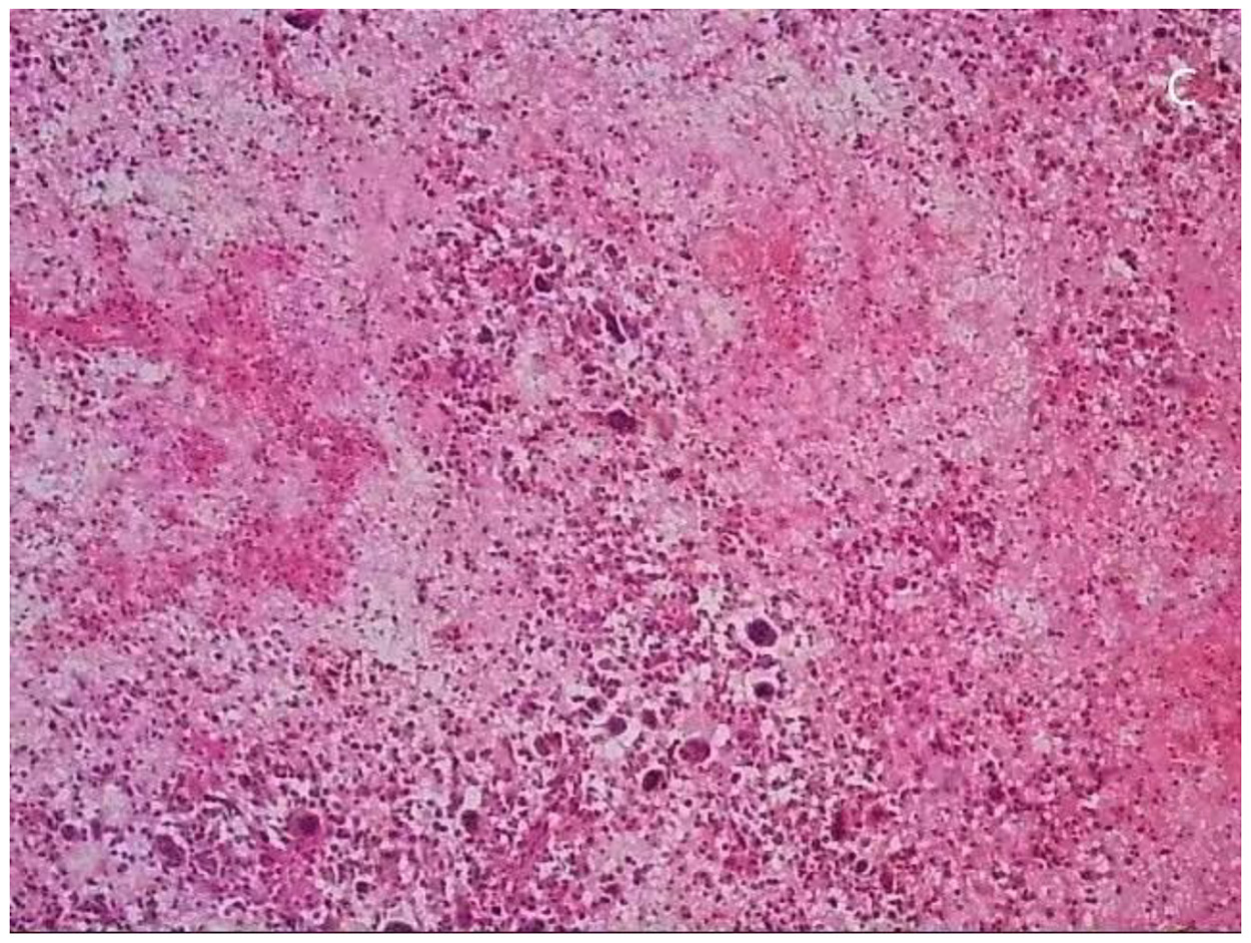
HE staining of tumors (X100).

**Figure 4.**
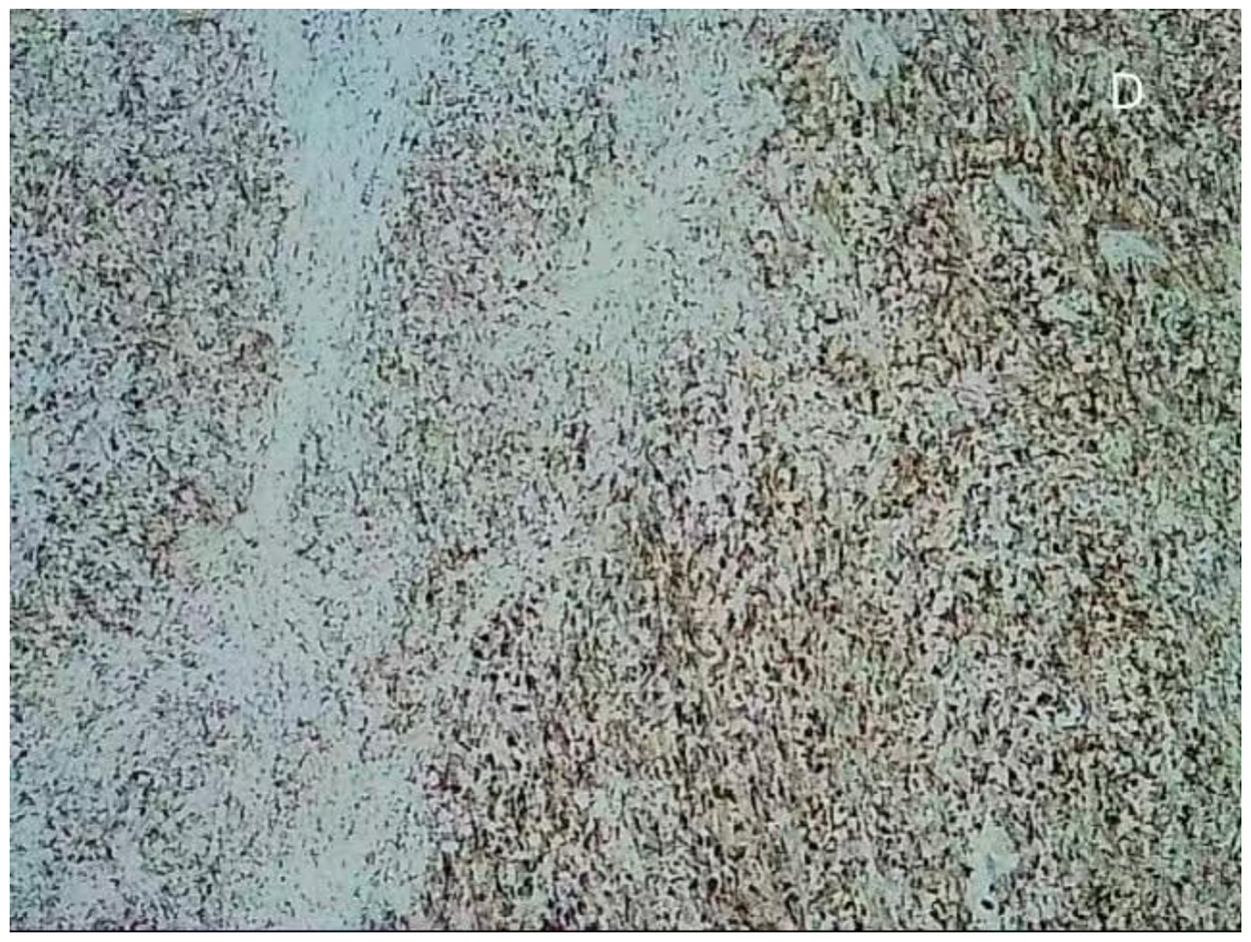
Tumor immunohistochemical staining.

**Figure 5.**
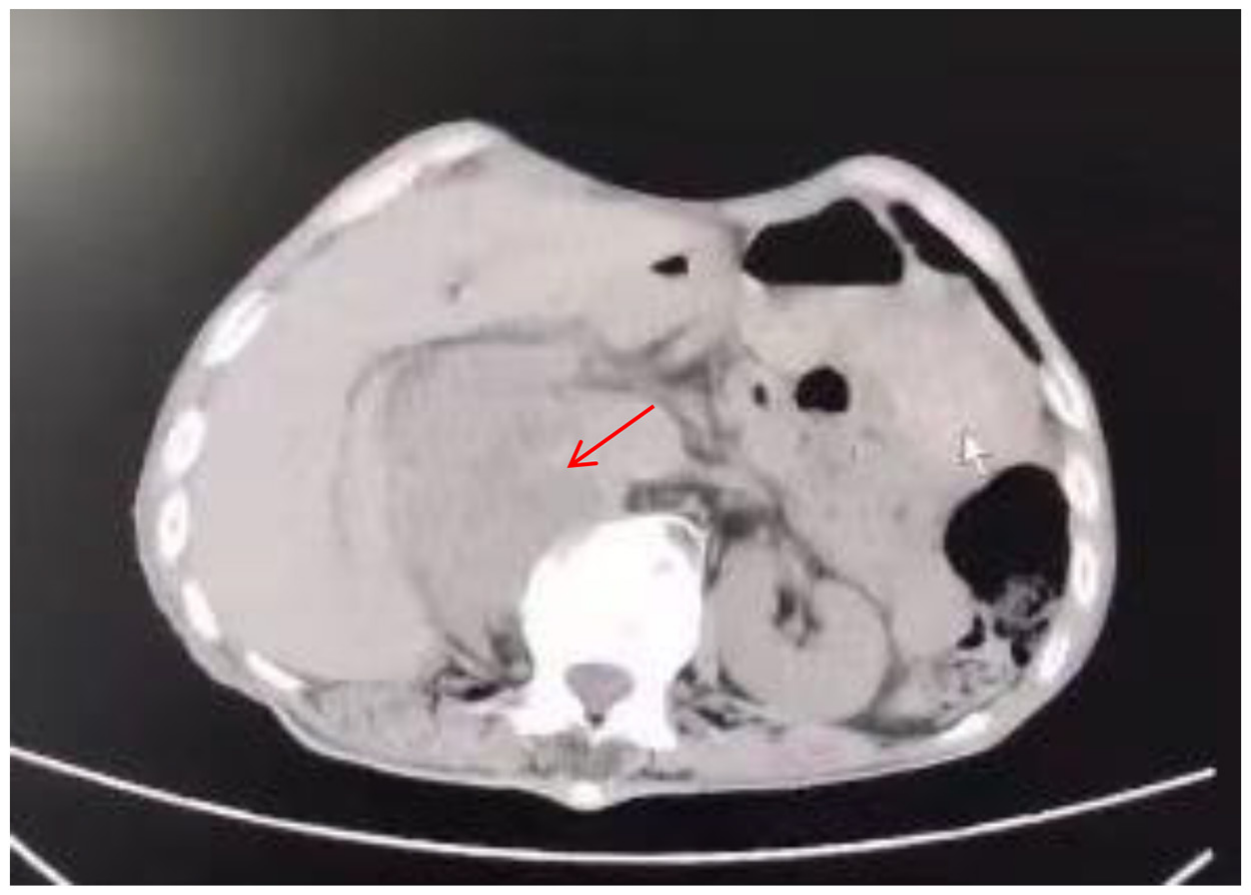
Follow-up abdominal CT shows adrenal metastasis. CT = computed tomography.

**Figure 6.**
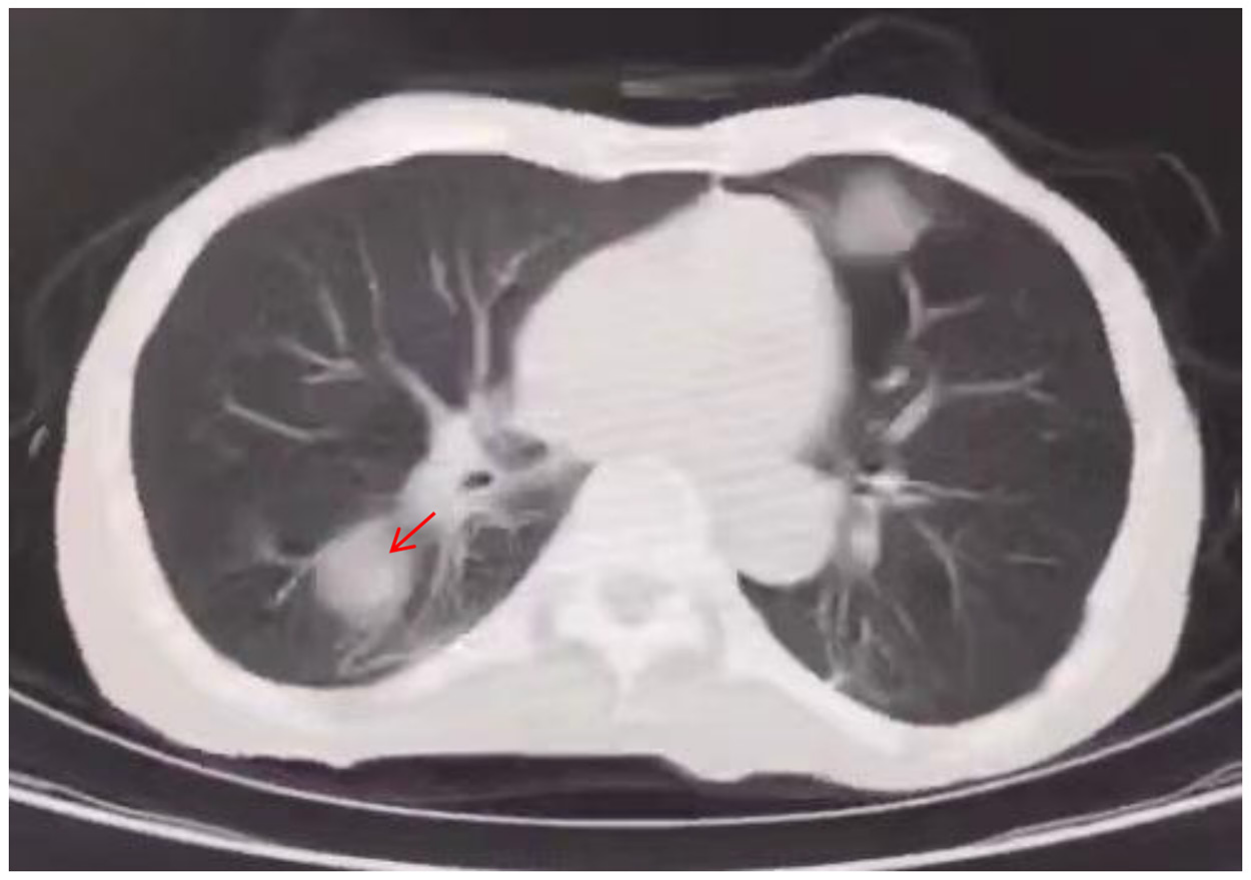
Follow-up chest CT shows lung metastasis. CT = computed tomography.

A 72-year-old man was admitted to the hospital with a 3-day history of right-sided low back pain. The patient had symptoms of hematuria and painful urination. Urinalysis showed an erythrocyte count of 15,312.0/μL and a leukocyte count of 276.0/μL. Computed tomography urography showed a thickened and rigid wall of the right pelvic-ureteral migratory segment, with a localized mass of approximately 3.3 × 2.7 cm, with heterogeneous density and uneven enhancement on enhancement scanning, with compression of the right renal pelvis, and no obvious enlarged lymph nodes in the retroperitoneum. The right renal pelvis was compressed, and no obvious enlarged lymph nodes were seen in the retroperitoneum (Figs. 1 and 2). Before surgery, the possibility of renal pelvis tumor was considered, and the whole right kidney and right ureter were surgically removed. Postoperative pathology showed a tumor rich in giant cells, consistent with GCT-ST. The ureteral segment was negative. Immunohistochemistry: tumor cells CD68 (+), CD163 (+), Langerin (−), SMA (−), P63 (−), SATB-2 (−), S100 (−), Des (−) (Figs. 3 and 4). Six months after the patient recovered and was discharged from the hospital, a follow-up examination suggested adrenal and lung metastases (Figs. 5 and 6), the patient abandoned further treatment, and the patient died 1 year and 3 months after the operation.

## 3. Discussion

Giant cell tumors are categorized as either GCTB or giant cell tumor of soft tissue. GCTB is classified as a locally aggressive neoplasm with intermediate malignant potential by the World Health Organization, owing to its high recurrence rate and propensity for pulmonary metastasis.^[3]^ Conversely, GCT-ST is a rare soft tissue neoplasm histopathologically similar to osteoclast-rich giant cell tumors of bone. Soft tissue giant cell tumor predominantly arises in the thigh, parotid gland, or subcutaneous tissues, with occasional occurrences in the head, neck, or mediastinum.^[4–6]^ Reports of GCT-ST originating from renal pelvic tissue are exceedingly uncommon. In this case, the patient was diagnosed with a GCT-ST of the renal pelvis, which subsequently metastasized to the adrenal gland and lungs within 6 months postradical nephrectomy. The patient ultimately succumbed in September after discontinuing treatment independently.

Giant cell tumor of soft tissue represents an exceedingly rare neoplastic entity. Initially characterized in 1972 by Slam and Sissons, who documented ten cases of soft tissue-origin tumors morphologically akin to osteoblastic giant cell tumors, traditionally regarded as benign.^[7]^ In the same year, Guccion et al^[8]^ reported thirty-two cases exhibiting prominent osteoblastic giant cells, demonstrating aggressive histopathological features and biological behavior, yet later identified as resembling what was then classified as malignant fibrous histiocytomas. Presently, GCT-ST is classified as a tumor with low malignant potential, and according to the World Health Organization 2013 classification, it is designated as a fibrohistiocytic tumor of intermediate (occasionally metastatic) grade, characterized as a giant cell tumor arising in soft tissue, with clinical and histological features similar to osseous counterparts, and infrequently exhibiting metastatic spread.

Oliveira et al^[9]^ documented 22 instances of giant cell tumor of soft tissue, with 16 cases undergoing clinical follow-up; among these, only 1 exhibited recurrence and pulmonary metastasis, ultimately resulting in mortality due to the neoplasm. Dodd et al^[10]^ reported a case of malignant GCT originating in the lower extremity, with subsequent pulmonary metastasis following local surgical excision. These findings indicate that GCT-ST possesses potential malignant transformation, predominantly exhibiting benign histopathology, although malignant variants are indeed observed.

Giant cell tumor of soft tissue predominantly manifests in the superficial soft tissues of the extremities, trunk, and head and neck regions. It is frequently situated subcutaneously or involves the integumentary structures or superficial fascia, with occasional infiltration into deeper soft tissues. Renal pelvis involvement is exceedingly rare. Gross examination reveals a nodular, solid, reddish-brown mass with a gravelly, well-circumscribed cut surface. Histologically, the lesion comprises a collagenous stroma dividing the nodule, composed of mononuclear cells with round to oval nuclei, alongside multinucleated osteoclast-like giant cells exhibiting nuclei comparable to those of mononuclear cells, with nuclear counts reaching up to dozens per cell. Nuclear atypia is minimal, with a mitotic rate of approximately 1 to 30 per 10 high-power fields. The tumor demonstrates uniform cellular morphology without significant heterogeneity or pleomorphism; tumor giant cells are absent, and necrosis is infrequent. Chemotactic infiltration is observed in approximately 50% of cases. Immunohistochemically, multinucleated giant cells show strong positivity for CD163 and CD68, whereas monocytes display weak expression in select cases. The neoplastic cells generally lack cytokeratin (CK) and S-100 expression, with occasional focal positivity. These features are consistent with the current case.

The histogenesis of GCT-ST remains uncertain; it is unclear whether osteoclast-like giant cells represent an atypical reactive process or a divergent differentiation pathway of the tumor, raising questions about their epithelial or histiocytic origin. Potential cellular sources include undifferentiated mesenchymal cells, epithelial cells, and histiocytes. Most studies suggest that extraosseous osteoclast-like giant cells derive from the monocyte-macrophage lineage, capable of multinucleation, as demonstrated through light microscopy, electron microscopy, and immunohistochemistry.^[11]^ Gaspar et al^[12]^ observed numerous lysosomal granules within monocyte cytoplasm via electron microscopy, supporting a monocyte-macrophage origin. Additionally, electron microscopy detected surface interactions between mast cells, eosinophils, and monocyte membranes, although the significance of this phenomenon remains to be elucidated, potentially offering avenues for further research.

Due to the rarity of GCT-ST, the patient’s initial hospitalization diagnosis did not sufficiently consider GCT-ST as a differential, resulting in diagnostic challenges and potential misdiagnosis. Differentiation of GCT-ST relies primarily on histopathological evaluation, with particular emphasis on relevant differential diagnostic factors: Extraosseous giant cell tumor, Urothelial carcinoma of the renal pelvis (transitional cell carcinoma), Pyelitis or infectious lesions, and Benign renal pelvis lesions (such as papillomas or fibroepithelial polyps).

In summary, giant cell tumor of soft tissue occurring within the renal pelvis represents a rare neoplasm, with limited case reports and a lack of awareness among clinicians regarding differential diagnosis. Definitive diagnosis relies on histopathological examination. Clinicians should include GCT-ST in the differential diagnosis of renal pelvic masses, and when feasible, en bloc surgical resection remains the optimal therapeutic approach. As demonstrated in this case, rapid postoperative recurrence and metastasis necessitate vigilant follow-up; strict postoperative surveillance is essential. Upon detection of metastatic spread, multidisciplinary oncological consultation should be pursued to formulate a coordinated treatment plan.

## Author contributions

**Conceptualization:** Yong Ou, Zhi-gang Chen, Mao-lin Ye, Huasong Hua.

**Data curation:** Yong Ou.

**Formal analysis:** Yong Ou.

**Methodology:** Ming-xian Chen, Kai Wang.

**Writing – original draft:** Hua-kang Wang, Hua Yang.

**Writing – review & editing:** Yun Hu, Zhong Zheng.
